# Supplementary material for: The Effect of Endurance and Endurance-Strength Training on Bone Health and Body Composition in Centrally Obese Women—A Randomised Pilot Trial
Source: Healthcare (Basel). 2022 Apr 28;10(5):821. doi: 10.3390/healthcare10050821 (PMC9140472; doi:10.3390/healthcare10050821)
Supplement: Supplementary file 1 [file healthcare-10-00821-s001.zip › Table S2.pdf]

Table S2. Scheme of strength exercises.

Day of the week	Exercise	Number of series/repetitions
Monday	1. Push the barbell in front of chest upwards while standing.<br>2. One minute recovery break.<br>3. Push the barbell up from behind head in a standing position.<br>4. One minute recovery break.<br>5. Bend elbow joints through their full range of motion while standing.	3/16*
Wednesday	1. Kneel straight on a bean type balance ball (across on the bean) – maintaining balance on unstable ground.<br>2. Lie on your stomach on a bean type balance ball (along the bean ball) – maintain body on the ball so that no part of body touches the ground.<br>3. Sit astride on a bean type balance ball so that no part of your body touches the ground.	depending on fitness level
Friday	1. Do squats with a barbell on shoulders to the right angle, at a medium pace counted on "3".	6/10
	2. Do squats with a barbell on shoulders to the right angle with varying pace in the following sequence: 1 slow, 1 quick … 5 slow, 5 quick	6/30
	3. Perform isometric contractions while doing a squat (90° knee bend), with varying duration. The sequence is: 10 s, 15 s, 20 s, 25 s, 30 s, 25 s, 20 s, 15 s, 10 s.	9

*Series interrupted with an approximately. 10-12 s isometric contraction in intermediate position (approx. 90° bend at the elbow joint).
